# Supplementary material for: Unveiling the Bioactive Potential of the Invasive Jellyfish Phyllorhiza punctata Through Integrative Transcriptomic and Proteomic Analyses
Source: Biomolecules. 2025 Aug 4;15(8):1121. doi: 10.3390/biom15081121 (PMC12383608; doi:10.3390/biom15081121)
Supplement: Supplementary file 1 [file biomolecules-15-01121-s001.zip › Suplementary Material/FileS3/PEPFOLD4/XP_065070279_16095/clusters.html]

 PEP-FOLD clusters


Download archive of all models  here   

|  |  |  |
| --- | --- | --- |
| # | file | sOPEP |
| # cluster 1 - Size: 2 |  |  |
|  | XP\_065070279\_16095-model1 | -6.32618 |
|  | XP\_065070279\_16095-model1.1 | -3.50926 |
| # cluster 2 - Size: 16 |  |  |
|  | XP\_065070279\_16095-model2 | -5.32874 |
|  | XP\_065070279\_16095-model2.1 | -5.25573 |
|  | XP\_065070279\_16095-model2.2 | -5.18221 |
|  | XP\_065070279\_16095-model2.3 | -5.17411 |
|  | XP\_065070279\_16095-model2.4 | -5.14006 |
|  | XP\_065070279\_16095-model2.5 | -5.11324 |
|  | XP\_065070279\_16095-model2.6 | -5.03915 |
|  | XP\_065070279\_16095-model2.7 | -5.02098 |
|  | XP\_065070279\_16095-model2.8 | -5.00017 |
|  | XP\_065070279\_16095-model2.9 | -4.61748 |
|  | XP\_065070279\_16095-model2.10 | -4.56084 |
|  | XP\_065070279\_16095-model2.11 | -4.55419 |
|  | XP\_065070279\_16095-model2.12 | -4.43525 |
|  | XP\_065070279\_16095-model2.13 | -4.36417 |
|  | XP\_065070279\_16095-model2.14 | -4.09777 |
|  | XP\_065070279\_16095-model2.15 | -4.03134 |
| # cluster 3 - Size: 2 |  |  |
|  | XP\_065070279\_16095-model3 | -5.28487 |
|  | XP\_065070279\_16095-model3.1 | -3.62612 |
| # cluster 4 - Size: 2 |  |  |
|  | XP\_065070279\_16095-model4 | -5.22971 |
|  | XP\_065070279\_16095-model4.1 | -5.13248 |
| # cluster 5 - Size: 3 |  |  |
|  | XP\_065070279\_16095-model5 | -5.21579 |
|  | XP\_065070279\_16095-model5.1 | -3.94217 |
|  | XP\_065070279\_16095-model5.2 | -3.69424 |
| # cluster 6 - Size: 9 |  |  |
|  | XP\_065070279\_16095-model6 | -4.93011 |
|  | XP\_065070279\_16095-model6.1 | -4.64909 |
|  | XP\_065070279\_16095-model6.2 | -4.34666 |
|  | XP\_065070279\_16095-model6.3 | -4.20057 |
|  | XP\_065070279\_16095-model6.4 | -4.09426 |
|  | XP\_065070279\_16095-model6.5 | -3.95015 |
|  | XP\_065070279\_16095-model6.6 | -3.90744 |
|  | XP\_065070279\_16095-model6.7 | -3.90294 |
|  | XP\_065070279\_16095-model6.8 | -3.66671 |
| # cluster 7 - Size: 1 |  |  |
|  | XP\_065070279\_16095-model7 | -4.87969 |
| # cluster 8 - Size: 5 |  |  |
|  | XP\_065070279\_16095-model8 | -4.41098 |
|  | XP\_065070279\_16095-model8.1 | -4.32326 |
|  | XP\_065070279\_16095-model8.2 | -4.2658 |
|  | XP\_065070279\_16095-model8.3 | -4.03834 |
|  | XP\_065070279\_16095-model8.4 | -3.93688 |
| # cluster 9 - Size: 6 |  |  |
|  | XP\_065070279\_16095-model9 | -4.19617 |
|  | XP\_065070279\_16095-model9.1 | -4.15579 |
|  | XP\_065070279\_16095-model9.2 | -4.14611 |
|  | XP\_065070279\_16095-model9.3 | -4.05519 |
|  | XP\_065070279\_16095-model9.4 | -3.58997 |
|  | XP\_065070279\_16095-model9.5 | -3.54164 |
| # cluster 10 - Size: 1 |  |  |
|  | XP\_065070279\_16095-model10 | -4.09815 |
| # cluster 11 - Size: 2 |  |  |
|  | XP\_065070279\_16095-model11 | -4.0058 |
|  | XP\_065070279\_16095-model11.1 | -3.97719 |
| # cluster 12 - Size: 1 |  |  |
|  | XP\_065070279\_16095-model12 | -3.91841 |
| # cluster 13 - Size: 3 |  |  |
|  | XP\_065070279\_16095-model13 | -3.91491 |
|  | XP\_065070279\_16095-model13.1 | -3.5362 |
|  | XP\_065070279\_16095-model13.2 | -3.45335 |
| # cluster 14 - Size: 5 |  |  |
|  | XP\_065070279\_16095-model14 | -3.88422 |
|  | XP\_065070279\_16095-model14.1 | -3.47458 |
|  | XP\_065070279\_16095-model14.2 | -3.41191 |
|  | XP\_065070279\_16095-model14.3 | -3.37578 |
|  | XP\_065070279\_16095-model14.4 | -3.36961 |
| # cluster 15 - Size: 1 |  |  |
|  | XP\_065070279\_16095-model15 | -3.86315 |
| # cluster 16 - Size: 2 |  |  |
|  | XP\_065070279\_16095-model16 | -3.81732 |
|  | XP\_065070279\_16095-model16.1 | -3.31574 |
| # cluster 17 - Size: 2 |  |  |
|  | XP\_065070279\_16095-model17 | -3.79389 |
|  | XP\_065070279\_16095-model17.1 | -3.39934 |
| # cluster 18 - Size: 1 |  |  |
|  | XP\_065070279\_16095-model18 | -3.71813 |
| # cluster 19 - Size: 2 |  |  |
|  | XP\_065070279\_16095-model19 | -3.6615 |
|  | XP\_065070279\_16095-model19.1 | -3.47298 |
| # cluster 20 - Size: 2 |  |  |
|  | XP\_065070279\_16095-model20 | -3.64764 |
|  | XP\_065070279\_16095-model20.1 | -3.48805 |
| # cluster 21 - Size: 2 |  |  |
|  | XP\_065070279\_16095-model21 | -3.64334 |
|  | XP\_065070279\_16095-model21.1 | -3.53588 |
| # cluster 22 - Size: 1 |  |  |
|  | XP\_065070279\_16095-model22 | -3.6216 |
| # cluster 23 - Size: 2 |  |  |
|  | XP\_065070279\_16095-model23 | -3.61358 |
|  | XP\_065070279\_16095-model23.1 | -3.29652 |
| # cluster 24 - Size: 3 |  |  |
|  | XP\_065070279\_16095-model24 | -3.59438 |
|  | XP\_065070279\_16095-model24.1 | -3.55481 |
|  | XP\_065070279\_16095-model24.2 | -3.54411 |
| # cluster 25 - Size: 7 |  |  |
|  | XP\_065070279\_16095-model25 | -3.54261 |
|  | XP\_065070279\_16095-model25.1 | -3.49568 |
|  | XP\_065070279\_16095-model25.2 | -3.46432 |
|  | XP\_065070279\_16095-model25.3 | -3.44512 |
|  | XP\_065070279\_16095-model25.4 | -3.44402 |
|  | XP\_065070279\_16095-model25.5 | -3.43864 |
|  | XP\_065070279\_16095-model25.6 | -3.42677 |
| # cluster 26 - Size: 2 |  |  |
|  | XP\_065070279\_16095-model26 | -3.51949 |
|  | XP\_065070279\_16095-model26.1 | -3.43237 |
| # cluster 27 - Size: 1 |  |  |
|  | XP\_065070279\_16095-model27 | -3.51674 |
| # cluster 28 - Size: 2 |  |  |
|  | XP\_065070279\_16095-model28 | -3.49482 |
|  | XP\_065070279\_16095-model28.1 | -3.37134 |
| # cluster 29 - Size: 2 |  |  |
|  | XP\_065070279\_16095-model29 | -3.49029 |
|  | XP\_065070279\_16095-model29.1 | -3.3735 |
| # cluster 30 - Size: 2 |  |  |
|  | XP\_065070279\_16095-model30 | -3.47846 |
|  | XP\_065070279\_16095-model30.1 | -3.43479 |
| # cluster 31 - Size: 1 |  |  |
|  | XP\_065070279\_16095-model31 | -3.42827 |
| # cluster 32 - Size: 2 |  |  |
|  | XP\_065070279\_16095-model32 | -3.41917 |
|  | XP\_065070279\_16095-model32.1 | -3.40667 |
| # cluster 33 - Size: 2 |  |  |
|  | XP\_065070279\_16095-model33 | -3.41887 |
|  | XP\_065070279\_16095-model33.1 | -3.41319 |
| # cluster 34 - Size: 1 |  |  |
|  | XP\_065070279\_16095-model34 | -3.41247 |
| # cluster 35 - Size: 1 |  |  |
|  | XP\_065070279\_16095-model35 | -3.31759 |
| # cluster 36 - Size: 1 |  |  |
|  | XP\_065070279\_16095-model36 | -3.29614 |
|  |  |  |
